# Bioengineering of Cytochrome P450 OleT_JE_: How Does Substrate Positioning Affect the Product Distributions?

**DOI:** 10.3390/molecules25112675

**Published:** 2020-06-09

**Authors:** Fabián G. Cantú Reinhard, Yen-Ting Lin, Agnieszka Stańczak, Sam P. de Visser

**Affiliations:** 1 Manchester Institute of Biotechnology and Department of Chemical Engineering and Analytical Science, The University of Manchester, 131 Princess Street, Manchester M1 7DN, UK; fabian.cantureinhard@manchester.ac.uk (F.G.C.R.); yen-ting.lin@manchester.ac.uk (Y.-T.L.); agasami@wp.pl (A.S.); 2 Faculty of Chemistry, Silesian University of Technology, 44-100 Gliwice, Poland

**Keywords:** density functional theory, enzyme catalysis, enzyme mechanism, QM/MM methods, heme enzymes, bioengineering, iron, heme, compound I

## Abstract

The cytochromes P450 are versatile enzymes found in all forms of life. Most P450s use dioxygen on a heme center to activate substrates, but one class of P450s utilizes hydrogen peroxide instead. Within the class of P450 peroxygenases, the P450 OleT_JE_ isozyme binds fatty acid substrates and converts them into a range of products through the α-hydroxylation, β-hydroxylation and decarboxylation of the substrate. The latter produces hydrocarbon products and hence can be used as biofuels. The origin of these product distributions is unclear, and, as such, we decided to investigate substrate positioning in the active site and find out what the effect is on the chemoselectivity of the reaction. In this work we present a detailed computational study on the wild-type and engineered structures of P450 OleT_JE_ using a combination of density functional theory and quantum mechanics/molecular mechanics methods. We initially explore the wild-type structure with a variety of methods and models and show that various substrate activation transition states are close in energy and hence small perturbations as through the protein may affect product distributions. We then engineered the protein by generating an in silico model of the double mutant Asn242Arg/Arg245Asn that moves the position of an active site Arg residue in the substrate-binding pocket that is known to form a salt-bridge with the substrate. The substrate activation by the iron(IV)-oxo heme cation radical species (Compound I) was again studied using quantum mechanics/molecular mechanics (QM/MM) methods. Dramatic differences in reactivity patterns, barrier heights and structure are seen, which shows the importance of correct substrate positioning in the protein and the effect of the second-coordination sphere on the selectivity and activity of enzymes.

## 1. Introduction

The cytochromes P450 appear in most forms of life and generally act as monoxygenases for vital biotransformations, whereby they utilize molecular oxygen on a heme center and transfer one of the oxygen atoms of O_2_ to substrate, while the other oxygen atom is reduced to a water molecule [1,2,3,4,5,6,7,8,9]. In the human body, the P450s are found in many organs and particularly in the liver, where they are involved in the biosynthesis of hormones, including serotonin and estrogen [10,11]. In addition, they take part in the biodegradation of drug molecules and xenobiotics, which makes them a key target for the pharmaceutical industry [12]. The P450 reactivity is highly versatile, but the key mechanism involves the activation of inert C‒H bonds into alcohols [13,14,15]; although many reactions show high stereo- and regioselectivity.

**Scheme 1:**
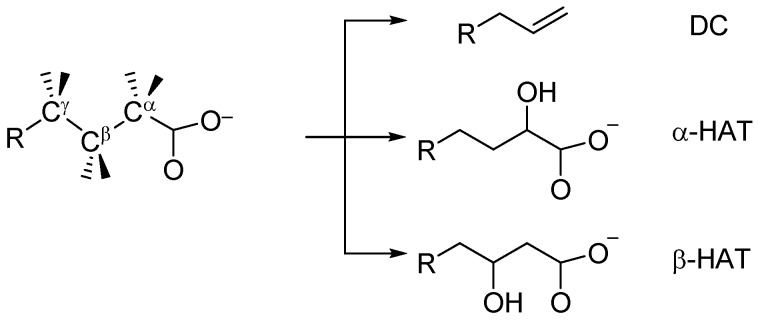
Reaction products observed for fatty acid activation by P450 OleT_JE_.

Applications of P450 enzymes in biocatalysis and biotechnology have been limited so far as they require redox partners and external protons in their catalytic cycle. A recently discovered class of P450 isozymes, however, uses hydrogen peroxide rather than O_2_ and reacts as a peroxygenase with substrates [16,17]. As such, these P450 peroxygenases do not need a redox partner and have better applicability in biotechnology. One specific P450 peroxygenase, namely OleT_JE_ from *Jeotgalicoccus sp* reacts with long-chain fatty acids through decarboxylation of the substrate to form terminal olefins, which may have applications in biofuel synthesis. Thus, P450 OleT_JE_ reacts with linear fatty acids on a heme active site, but the reaction is not selective and gives a number of products and by-products (Scheme 1) [18,19,20,21]. One pathway (labelled DC in Scheme 1) is known to lead to substrate decarboxylation and the formation of terminal olefin and CO_2_. Unfortunately, the decarboxylation yield varies with the substrate used and often is well below 80% [22,23,24], hence the enzyme has limited use in biotechnology for the biosynthesis of terminal olefins as biofuels. In addition, a significant amount of by-products is observed as a result of, e.g., α-hydroxylation (through C^α^‒H hydrogen atom transfer, α-HAT) and β-hydroxylation (β-HAT). Recently, a fourth pathway for fatty acid desaturation was identified that converts saturated fatty acids into unsaturated fatty acids through two consecutive hydrogen atom abstraction steps [25]. Thus, with myristic acid as a substrate, 71% decarboxylation and 29% β-hydroxylation by OleT_JE_ was obtained, whereas 2-methylbutyrate as a substrate gave 34% α-hydroxylation and 66% desaturation and no decarboxylation or β-hydroxylation products [25]. Interestingly, the P450 monoxygenase isozymes P450_BSβ_ and P450_SP_α utilize the same substrate sets but convert them to dominant β-HAT and α-HAT products, respectively [26,27,28]. Clearly, the substrate-binding pocket and substrate-binding orientation are important to guide the reaction to the wanted products. To gain further insight into how the substrate-binding position determines the product distributions, we decided to expand our computational study on OleT_JE_ and bioengineer the protein for optimal decarboxylation product distributions.

**Figure 1:**
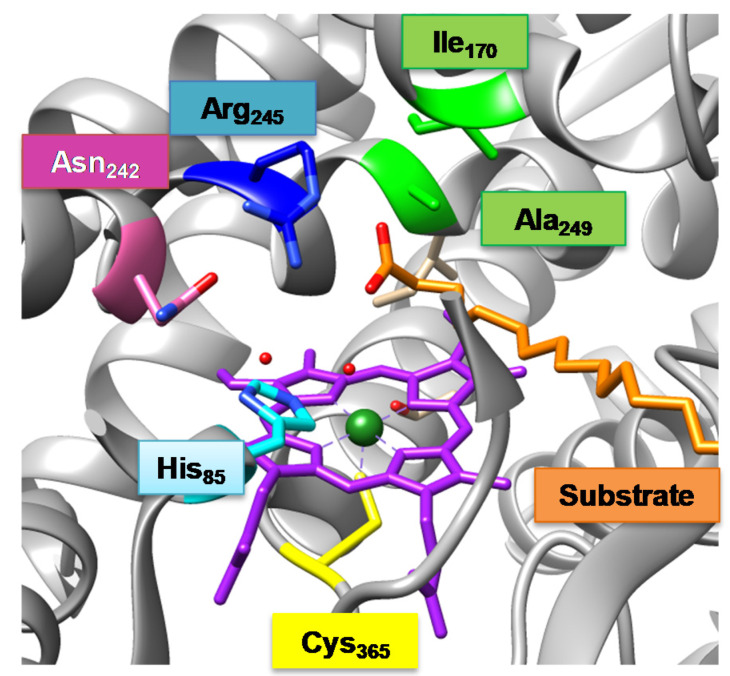
Active site structure of P450 OleT_JE_ as taken from the 4L40 pdb file.

The P450s contain a heme active site and a substrate-binding pocket that varies in shape and size among the P450 isozymes [29,30]. Figure 1 displays the active site region of P450 OleT_JE_ as taken from the 4L40 protein databank file (pdb) [31]. This is a substrate-bound resting state conformation with the heme iron(III) bound to the protein via a bond with the thiolate group of Cys_365_ on the axial site, while a water molecule fills the position on the distal site. The substrate is located in a channel on the distal site and is held in position through a salt bridge with an arginine residue (Arg_245_). A close inspection of the enzyme-substrate topology reveals that the Arg_245_-substrate salt bridge is the main drive for the substrate positioning, while aliphatic residues, such as Ile_170_ and Ala_249_, line the substrate-binding channel. Consequently, our efforts in bioengineering P450 OleT_JE_ were focused on mutants whereby the active site Arg residue was relocated to an alternative position.

**Scheme 2:**
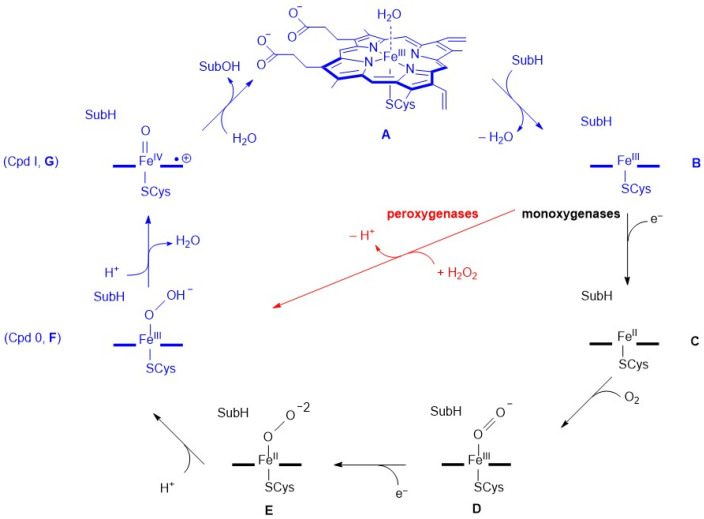
Catalytic cycle of P450 monoxygenases (pathways in blue and black) and P450 peroxygenases (pathways in blue and red). **A** is the resting state; **B** is the pentacoordinated iron(III) complex; **C** is the pentacoordinated iron(II) complex; **D** is the iron(III)-superoxo state; **E** is the iron(II)-peroxo intermediate; **F** is Cpd 0; **G** is Cpd I.

The catalytic cycle of P450 monoxygenases and peroxygenases is schematically depicted in Scheme 2. Both cycles start from the resting state (**A**), where the heme iron is ligated to a solvent water molecule and linked to the protein through the cysteinate axial ligand. Substrate (SubH) binding into the active site, displaces the distal water molecule to form the pentacoordinated iron(III) complex **B**. In typical P450 monoxygenases [1,2,3,4,5,6,7,8,9,12,] the heme picks up an electron from the redox partner (P450 reductase) to form the pentacoordinated iron(II)-heme (**C**), which binds molecular oxygen to form structure **D**. The latter is reduced to form structure **E** and is protonated to give Compound 0 (Cpd 0), the iron(III)-hydroperoxo complex **F**. A final protonation leads to cleavage of the O‒O bond and the formation of Compound I (Cpd I), which is an iron(IV)-oxo heme cation radical species, structure **G**. The latter reacts with the substrate through hydrogen atom abstraction and OH rebound to form alcohol products (SubOH) and rebinds a water molecule to return to the resting state.

For P450 monoxygenases there is ample evidence of the catalytic cycle and various intermediates have been trapped and characterized, including Cpd I [32]. Furthermore, the computational modelling of the catalytic cycle of P450 monoxygenases using either density functional theory (DFT) or quantum mechanics/molecular mechanics (QM/MM) converged to the same conclusion [32,33,34,35]. The catalytic cycle of P450 peroxygenases shows significant overlap with the monoxygenases and also starts with substrate-binding and the release of the water ligand from the heme. However, instead of a reduction of the heme, there is no reduction partner available but hydrogen peroxide binds and, through the release of a proton, is converted directly to the iron(III)-hydroperoxo species Cpd 0 through the peroxide-shunt pathway [32]. From that point, the cycle follows the same pathway as the monoxygenases by formation of Cpd I.

As many controversies remain on the catalytic cycle of P450 peroxygenases and how substrate positioning affects the product distributions, we decided to do a detailed computational study using density functional theory and QM/MM methods. The work shows that substrate positioning affects the kinetics and reactivity of the enzyme dramatically.

## 2. Results and Discussion

As mentioned in the introduction, the native P450 OleT_JE_ enzyme produces significant amounts of unwanted by-products and some of those originate from hydrogen atom abstraction from the C^α^-position of the substrate. We decided to model the reaction mechanism of fatty acid activation by P450 OleT_JE_ wild-type and mutant structures. We, therefore, started the work with a gas-phase study on a model complex and predicted the ideal orientation of the substrate in the binding pocket from that model. Thereafter, we create an engineered enzyme model with the ideal conformation of the gas-phase structure implemented. To this end, we dock the substrate into the crystal structure coordinates and engineer the protein in such a way that the salt bridge with an active site Arg residue still can be formed but the position of the substrate has shifted for optimal substrate activation to lead to decarboxylation products. Finally, a quantum mechanics/molecular mechanics (QM/MM) study on the hydrogen atom abstraction steps for wildtype (WT) and mutant structures was performed.

### 2.1. DFT Model Complexes on the Hydrogen Atom Abstraction Step by P450 OleT_JE_

All initial efforts presented here were focused on improving the regioselectivity of the first hydrogen atom transfer (HAT) by CpdI. Specifically, the effects of substrate-positioning on the reactivity and selectivity of the reaction was studied using a simplified gas-phase model complex. The hydrogen atom transfer from the substrate to CpdI was studied from the α-, β- and γ-positions of the substrate and activation energies were determined. We started with a minimal model that includes the heme abbreviated as protoporphyrin IX and all side-chains replaced by hydrogen atoms. In addition, the axial cysteinate was mimicked as thiolate as it was shown previously to compare well with the real system [36,37,38]. This simplified model excludes the steric, dispersion and electronic contributions of the enzyme in comparison to a full enzymatic structure.

**Figure 2:**
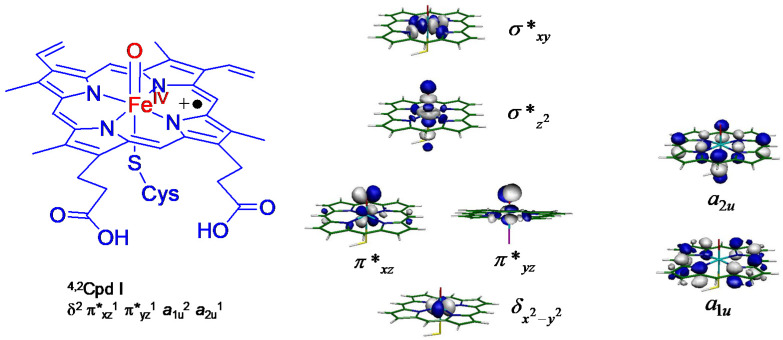
Electronic orbitals of doublet and quartet Cpd I with orbital occupation.

To understand the reactivity patterns, we display in Figure 2 a Cpd I structure and the high-lying occupied and low-lying virtual orbitals relevant for the reaction mechanisms. Thus, Cpd I is a high-valent structure formally with an iron(IV) inside an oxidized heme and ligated to an oxo dianion species. This cluster is overall charge neutral and its properties are determined by the metal 3d-orbitals and their interactions with ligand atoms. The right-hand-side of Figure 2 gives the relevant Cpd I orbitals. From bottom-to-top, the metal-type orbitals are the δ_x2-y2_, π*_xz_, π*_yz_, σ*_z2_ and σ*_xy_ orbitals. The δ-orbital is non-bonded in the plane of the porphyrin, the *xy* plane, and is doubly occupied. Higher lying is a pair of π* orbitals for the anti-bonding interactions between the iron and oxo groups (π*_xz_ and π*_yz_); both are singly occupied molecular orbitals in Cpd I. Finally, there are two virtual σ* orbitals along the *z*-axis (σ*_z2_) and in the heme plane (σ*_xy_). On the right-hand side of Figure 2, we show two high-lying porphyrin orbitals that in D_4h_ symmetry with the labels *a*_1u_ and *a*_2u_; the latter is singly occupied in Cpd I. Overall, therefore, both quartet and doublet spin states have the same orbital occupation, namely δ_x2-y2_^2^ *a*_1u_^2^ π*_xz_^1^ π*_yz_^1^ *a*_2u_^1^, whereby the *a*_2u_ radical is either up-spin or down-spin. As a consequence of the same orbital configuration, the two spin states are close in energy and usually calculated well within 1 kcal mol^‒1^. Previous DFT and QM/MM calculations confirmed these configurations as the ground state for Cpd I [32,33,34,35,36,37,38,39,40,41].

As Cpd I has two spin states that are close in energy, this implies there are two degenerate reactant species, namely doublet spin Cpd I and quartet spin Cpd I. Both spin states will react with substrate differently with different rate constants and sometimes even different reaction mechanisms. Therefore, P450 Cpd I shows two-state reactivity patterns [33,42], with a reaction on the doublet spin state surface and one on the quartet spin state surface. Often, in aliphatic hydroxylation or alkene epoxidation reactions, the two spin state surfaces are close in energy, particularly for the initial C‒H/C=C bond activation step [43,44,45,46,47,48,49,50,51,52,53,54,55]. By contrast, in aromatic hydroxylation or sulfoxidation reactions, where, typically, the reaction is determined by a two-electron process, the two spin states are significantly separated, thereby reducing the system to single-state-reactivity [56,57,58,59,60].

**Scheme 3:**
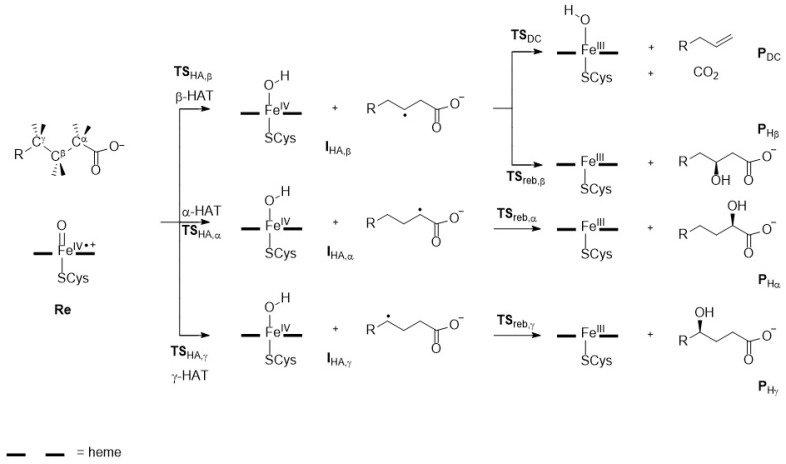
Reaction mechanism with the definition of transition states and local minima for fatty acid activation by P450 OleT_JE_.

Scheme 3 shows the overall mechanisms for the reactions of doublet and quartet CpdI with hexanoate substrate leading to hydroxylation of the C^α^ and C^β^ positions and decarboxylation and the definition of the structures along the reaction mechanisms. The reactions start with a hydrogen atom abstraction transition state from either the C^α^‒H, C^β^‒H or C^γ^‒H positions, designated **TS**_HA,α_, **TS**_HA,β_ and **TS**_HA,γ_, respectively, which lead to radical intermediates **I**_HA,α_, **I**_HA,β_ and **I**_HA,γ_. From the **I**_HA,α_ and **I**_HA,γ_ intermediates, the only pathway is OH rebound via transition state **TS**_reb_ to form the α- and γ-hydroxylated products **P**_Hα_ and **P**_Hγ_. By contrast, the mechanism from **I**_HA,β_ bifurcates and either leads to the decarboxylation products (**P**_DC_) via transition state **TS**_DC_ or alternatively via OH rebound (via transition state **TS**_reb,β_) to the, β-hydroxylated products **P**_H,β_. The spin state is given in superscript before the label for the individual doublet and quartet spin states.

The full mechanism shown in Scheme 3 was calculated with the small gas-phase DFT model, i.e., [Fe^IV^(O)(Por^+•^)(SH)]—^‒^OOCC^α^H_2_C,^β^ H_2_C_3_H_7_ with Por = protoporphyrin IX with all side chains along its periphery replaced by hydrogen atoms and thiolate as the axial ligand. To get a clear idea of what effects the enzyme has on the substrate activation energies, a preliminary simplified DFT study was performed on the initial steps of hydrogen atom abstraction from the α-, β- and γ-positions of the substrate. These results are then interpreted as the ideal model that has the ideal orientations of the substrate (hexanoic acid) and oxidant (Cpd I) in the gas phase. This model, obviously, excludes the enzyme’s steric and electronic contributions that determine the protein environment and substrate-binding pocket, so, by comparison (to a QM/MM model), one can discern the effects the enzyme is already imposing to the reactivity and structure. In the next stage, we engineer the full protein structure to try to predict what mutations might have a positive benefit on substrate positioning.

**Figure 3:**
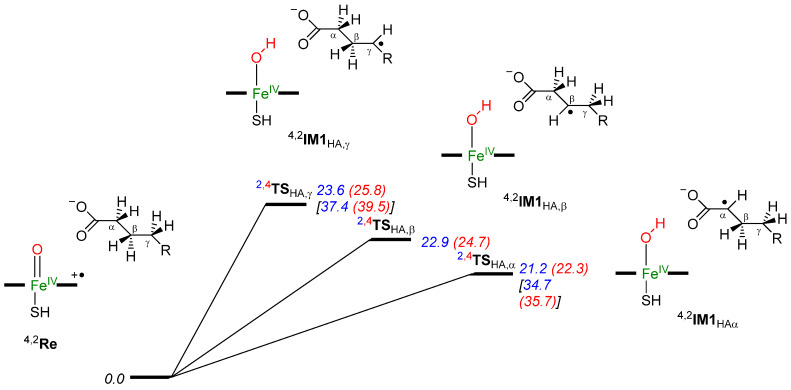
Potential energy landscape calculated at UB3LYP/BS1+ZPE//UB3LYP/BS2 for various hydrogen atom abstraction pathways by Cpd I of P450 OleT_JE_. Energies contain zero-point corrections and are given in kcal mol^‒1^. The values in square brackets are free energies with solvent, zero-point, entropic and thermal corrections at 298K included.

Figure 3 displays the potential energy landscape for the reactions for hydrogen atom abstraction from the α -, β- andγ -positions of the substrate, as these are the most likely and best accessible C‒H bonds in the substrate by Cpd I. As can be seen, the six hydrogen atom abstraction barriers for the three pathways are very similar in energy and range from 21.2 kcal mol^‒1^ for ^2^**TS**_HA,α_ to 25.8 kcal mol^‒1^ for ^4^**TS**_HA,γ_. Therefore, in the gas phase, all six hydrogen atom abstraction barriers seem accessible, where the C^α^ ‒H hydrogen atom abstraction pathway is predominant with the lowest two barriers, while the C^γ^ ‒H hydrogen atom abstraction is somewhat slower, and probably will lead to a smaller contribution of products. These trends stay the same regardless whether enthalpies or free energies are used. Previous work of our group [25,61], showed that substrate positioning is important, but in the enzymatic model with QM/MM and with arachidonic acid (*n*-C_19_H_40_COO^‒^) bound a favorable C^β^‒H hydrogen atom abstraction step. After this, the pathways bifurcate and lead to a mixture of decarboxylation and β-hydroxylation products. Note that the radical intermediates ^2,4^**I**_HA,β_ calculated here collapsed to decarboxylation products rapidly and no mechanism leading to β-hydroxylation could be identified. As such, the small model complexes show differences with respect to the enzymatic model, where substrate positioning affects barriers as well as mechanisms. The radical intermediates have a configuration of iron(IV)-hydroxo with a closed-shell heme and orbital occupation δ^2^ π*_xz_^1^ π*_yz_^1^ *a*_2u_^2^ f_Sub_^1^, where the latter orbital represents the radical on the substrate on the carbon atom that has lost its hydrogen atom.

**Figure 4:**
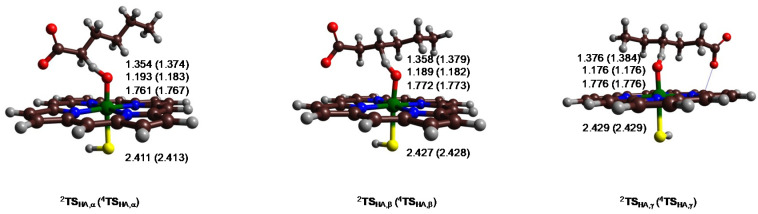
Optimized transition state geometries of the gas-phase density functional theory (DFT) pathways for hydrogen atom abstraction from the C^α^ ‒H, C^β^ ‒H and C ^γ^‒H positions. The bond lengths are in angstroms.

Optimized geometries of the six hydrogen atom abstraction transition states (**TS**_HA,α_, **TS**_HA,β_ and **TS**_HA,γ_) are shown in Figure 4. They are all very much alike and the only difference is the position of the substrate with respect to the iron-porphyrin system. The Fe‒S distances are long and range from 2.411 Å for ^2^**TS**_HA,α_ to 2.429 Å for the C^γ^‒H hydrogen atom abstraction barriers, while the Fe‒O distances are shortest for ^2^**TS**_HA,α_ (at 1.761 Å) and longest for both C^γ^‒H barriers. The O‒H distances are well shorter than the C‒H distances and hence the structures can be seen as product-like. In general, product-type are common for aliphatic hydrogen atom abstraction barriers [62,63] and the structures seen here match those found for activation of the secondary carbon atom in propane by a Cpd I model. This is not surprising, as the C‒H bond activation transition states in Figure 4 all are for secondary C‒H bonds.

Substrate decarboxylation is a two-step process, whereby first a hydrogen atom abstraction from the C^β^ ‒H group takes place prior to release of CO_2_ and electron transfer to the heme. However, the reaction can also proceed through β-hydroxylation by OH rebound instead. Recently, QM/MM calculations implicated that solvent molecules in the substrate-binding pocket may hinder the rebound step and guide the process to decarboxylation efficiently [61]. On the other hand, hydrogen atom abstraction from the C^α^ ‒H position cannot lead to decarboxylation as this is a high-energy pathway. Therefore, the key in the product selectivity process and the yield of olefin products lies in the initial hydrogen atom abstraction step, whereby hydrogen atom abstraction from the C^β^ ‒H position can give valuable products, i.e., hydrocarbons as biofuels, whereas hydrogen atom abstraction from the C^α^ ‒H will only give fatty acid hydroxylation. As such, we decided to investigate substrate-binding positions and bioengineer the protein in such a way that hydrogen atom abstraction from the C^α^ ‒H position is blocked while the one from the C^β^ ‒H position is enhanced.

### 2.2. DFT Model Complexes on Second Substrate Activation Step

**Scheme 4:**
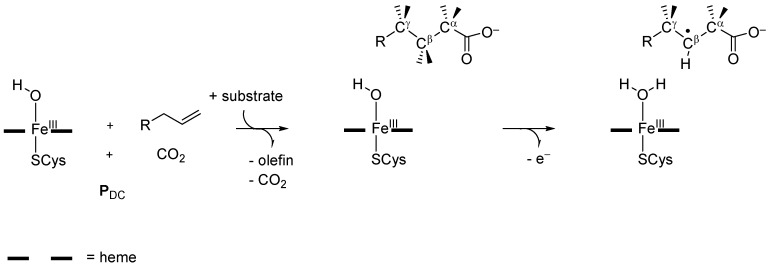
Alternative mechanism for second substrate activation during the catalytic cycle of P450 OleT_JE_.

A careful analysis of Scheme 3 above shows that the decarboxylation product complex **P**_DC_ gives an iron(III)-hydroxo, terminal olefin and CO_2_ products. However, this does not close the catalytic cycle, as the resting state is an iron(III)-water complex. In principle, a proton-relay mechanism could deliver a proton and convert the iron(III)-hydroxo to the resting state. Earlier work of ours though have shown that the iron(III)-hydroxo(heme) species is a reasonable oxidant of hydrogen atom abstraction processes of aliphatic groups [55] and, therefore, we hypothesized that it could react with another substrate through hydrogen atom abstraction. To test whether a new substrate could bind instead and do another cycle prior to returning to the resting state, we tested the mechanism shown in Scheme 4.

**Figure 5:**
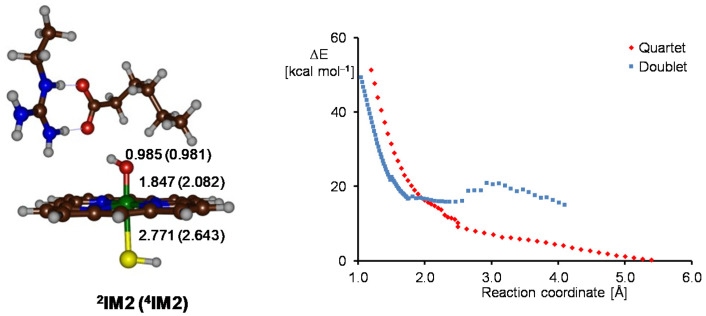
Optimized geometry of the iron(III)-hydroxo substrate complex of the catalytic cycle of P450 OleT_JE_ with bond lengths in angstroms. Geometry scan for C^β^‒H hydrogen atom abstraction in the quartet and doublet spin states.

We tested the second step in Scheme 4 using DFT model complexes containing [Fe^III^(OH)(Por)(SH)]^‒^ with hexanoate as substrate mimic and the methylguanidinium of Arg_245_ included as well. Optimized geometries of the iron(III)-hydroxo complex with substrate bound (^2,4^**IM2**) are shown on the left-hand-side of Figure 5. The Fe‒S bond lengths are very long (2.771 Å in ^2^**IM2** and 2.643 Å in ^4^**IM2**) and much longer than the structures shown before. This is as expected, as the metal oxidation state has decreased from iron(IV) in Cpd I to iron(III) in **IM2**. This results in a doublet electronic state with configuration δ^2^ π*_xz_^2^ π*_yz_^1^ or a quartet spin state with δ^2^ π*_xz_^1^ π*_yz_^1^ σ*_z2_^1^ [64]. Particularly, occupation of the σ*_z2_ orbital with one electron will elongate the Fe‒S and Fe‒O bonds dramatically as indeed seen from the optimized geometries in Figure 5. The difference in Fe‒O bond length between the doublet and quartet structures especially reflects this.

Next, the substrate activation by the iron(III)-hydroxo complexes in the doublet and quartet spin states was explored. To this end, a constraint geometry scan was run, whereby the C^β^ H‒O distance were fixed at specific distances and the rest of the geometry energy minimized. The doublet and quartet spin geometry scans for C^β^‒H hydrogen atom abstraction by ^2,4^**IM2** is shown on the right-hand side of Figure 5. In the quartet spin state, the curve is repulsive and never reaches a local minimum for a product complex, and, consequently, the quartet spin hydrogen atom abstraction is an unviable process. Interestingly, on the doublet spin state, the starting point is significantly higher in energy but, after a barrier of 20.7 kcal mol^‒1^, it settles into a local minimum with an energy about 15.8 kcal mol^‒1^ above reactants. This may be caused by the formation of a hydrogen bonding interaction between the substrate and OH group and also between the OH group and the Arg residue. Nevertheless, at even shorter distances, the curve is repulsive and no local minimum for an iron-water complex is found. Most probably, this process cannot happen without the release of an electron to the environment and the iron in our model cannot abstract it.

**Figure 6:**
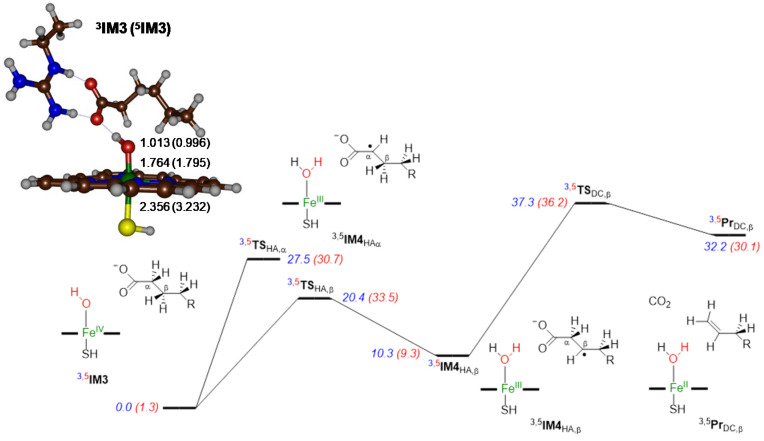
Optimized geometry of the iron(IV)-hydroxo substrate complex as potential intermediate in the catalytic cycle of P450 OleT_JE_ with bond lengths in angstroms. The potential energy landscape of substrate decarboxylation by ^3,5^**IM3** with energies (ΔE+ZPE values) in kcal mol^‒1^.

Subsequently, we tested the hypothesis, where the iron(III)-hydroxo species (**IM2**) is oxidized into an iron(IV)-hydroxo species (**IM3**) and then reacts with a new substrate. To this end, we removed an electron from ^2,4^**IM2** to get ^3,5^**IM3** or [Fe^IV^(OH)(Por)(SH)]^0^ and study the hydrogen atom abstraction from hexanoate substrate by **IM3** from the C^α^ ‒H and C^β^‒H positions. The pathway after C^β^‒H abstraction was followed to give decarboxylation products. Figure 6 displays the calculated potential energy profile and the optimized geometries of ^3,5^**IM3**. The removal of one electron from iron(III)-hydroxo to iron(IV)-hydroxo shortens the Fe‒S distance to 2.356Å in the triplet spin state but elongates the distance to 3.232Å in the quintet spin state. These distances relate to the molecular orbital occupations, which are δ^2^ π*_xz_^1^ π*_yz_^1^ in the triplet spin state and δ^1^ π*_xz_^1^ π*_yz_^1^ σ*_z2_^1^ in the quintet spin state. The occupation of the σ*_z2_ orbital with one electron elongates the Fe‒S distance as it is antibonding along that bond. Both spin states are close in energy, with a small preference for the triplet spin state. This spin-state ordering matches experimental and computational studies on hexacoordinated iron(IV)-hydroxo complexes in the literature that usually give a triplet spin ground state [5,9,55].

Next, we studied the mechanism leading to decarboxylation of substrate and calculated the hydrogen atom abstraction from the C^α^ ‒H and C^β^‒H positions in the triplet and quintet spin states. The lowest barrier is for C^β^‒H hydrogen atom abstraction in the triplet spin state at ΔE^‡^+ZPE = 20.4 kcal mol^‒1^. Significantly higher in energy are the hydrogen atom abstraction barriers from the C^α^‒H group with values of ΔE^‡^+ZPE = 27.5 kcal mol^‒1^ in the triplet spin state and ΔE^‡^+ZPE = 30.7 kcal mol^‒1^ in the quintet spin state. Even higher in energy is the barrier for the C^β^‒H hydrogen atom abstraction in the quintet spin state: ΔE^‡^+ZPE = 33.5 kcal mol^‒1^. Our calculated barriers, particularly for C^α^ ‒H hydrogen atom abstraction, implicate a very slow and even sluggish reaction.

The reaction pathway from radical intermediates ^3,5^**IM3**_HA,β_ to break the C‒C bond in the substrate, release CO_2_ and transfer an electron to the iron(III)-water group; however, it is high in energy and a decarboxylation barrier (via ^3,5^**TS**_DC,β_) of well over 35 kcal mol^‒1^ is found. Even the decarboxylation products are significantly higher in energy than reactants (by more than 30 kcal mol^‒1^); hence, this high endothermicity implicates that an iron(IV)-hydroxo species, even if it can be formed in the enzyme, will not be able to decarboxylate a second substrate molecule. Consequently, the catalytic cycle of P450 OleT_JE_ uses only one substrate and one H_2_O_2_ molecule per cycle. Moreover, the final reaction step will need to include a proton transfer from the solvent into the enzyme to convert the iron(III)-hydroxo complex into the resting state iron(III)-water form.

### 2.3. QM/MM calculations on WT enzyme

To supplement the DFT model calculations, we decided to explore full enzymatic structures using the QM/MM technique and used set-up procedures as previously discussed [65,66,67,68,69]. To this end, we took the crystal structure coordinates of the substrate-bound resting state of P450 OleT_JE_, i.e., 4L40 pdb file [31], and set it up for QM/MM calculations on wild-type and mutant structures. As the pdb file has an iron(III)-heme ligated to a water molecule in the sixth ligand position, we manually replaced it with an iron(IV)-oxo species with a starting Fe‒O distance of 1.63Å. In addition, we replaced the substrate by hexanoic acid by removing the aliphatic tail of arachidonic acid in the pdb. Hydrogen atoms were added using the PropKa software package [70] that assumes a pH = 7. In general, all Asp and Glu residues were deprotonated and all Arg and Lys residues protonated, while all histidine groups were kept as singly protonated. Key acidic and basic residues in the protein were manually inspected for their correct protonation states.

**Figure 7:**
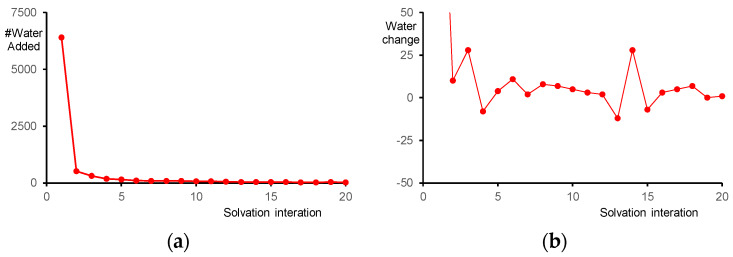
(**a**) Iterative solvation procedure during the set-up of wildtype Compound I structure of P450 OleT_JE_ with number of water molecules added per solvation step. (**b**) Change in the number of water molecules from one solvation step to the next.

Thereafter, an iterative solvation procedure was used, whereby solvent (water) was added to the protein and the system equilibrated for 100 ps in Charmm [71] at each step, while keeping the protein atoms fixed. The solvation procedure was repeated 20 times, see Figure 7 until only 30 water molecules were added in the cycle. Although a large number of water molecules were added in the first solvation cycle, namely 6406 water molecules; it is seen that after a few cycles the number of water molecules added drops steeply. Moreover, an analysis of the structures after solvation shows that few water molecules actually move into the protein. We then analyzed the number of water molecules that had entered the protein and counted the water molecules within a range of 20Å from the heme iron atom. The change in water molecules in the 20Å zone is displayed in Figure 7b. Thus, in the first solvation cycle, 211 water molecules enter the inner zone of 20Å from the heme iron atom. However, in the next cycle, only an additional 10 water molecules are seen to move into the 20Å zone. In the next 18 solvation cycles the number of water molecules in the 20Å zone around the heme iron atom fluctuates and the average number of water molecules that enters this zone in solvation steps 4 to 10 is about four water molecules. Furthermore, when we consider an inner zone of 10Å, an average of only one water molecule per solvation cycle is added over the full 20 solvation cycles. Therefore, a large number of solvation steps does not seem to increase the solvation levels in the inside of the protein dramatically and, probably, a handful of solvation cycles will be enough to get a reasonable solvated structure description.

**Figure 8:**
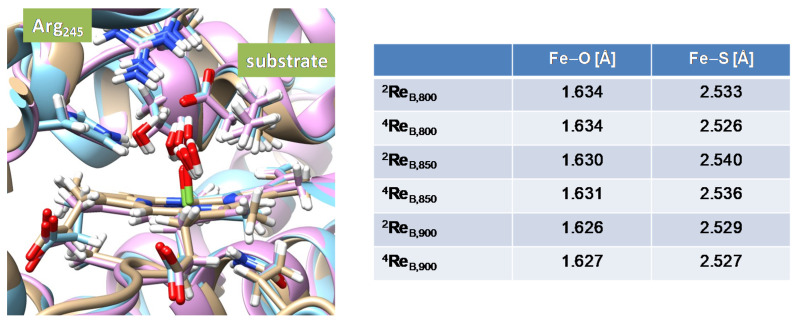
Quantum mechanics/molecular mechanics (QM/MM) optimized geometries of Compound I in the doublet and quartet spin states for snapshots Sn_800_, Sn_850_ and Sn_900_. Fe‒O and Fe‒S distances given in angstroms, while the left-hand-side gives an overlay of the doublet spin optimized structures.

Next, we took the fully solvated structure, heated it to room temperature and afterwards ran a 1ns molecular dynamics (MD) simulation using the Charmm forcefield [71]. Three random structures from the final stages of the MD simulation were selected as starting points for the QM/MM calculations, namely the snapshots after 800, 850 and 900ps: designated Sn_800_, Sn_850_ and Sn_900_. The QM region contains the heme without side chains, the substrate and the methylguanidine part of Arg_245_, while all the rest of the structure is in the MM region. The QM/MM optimized geometries of the doublet and quartet spin states of Compound I (CpdI), i.e., ^4,2^**Re**, are given in Figure 8. The top panel gives an overlay of the doublet spin optimized structures, i.e., ^2^**Re**_B,800_, ^2^**Re**_B,850_ and ^2^**Re**_B,900_. As can be seen, the three structures are very similar and most groups are in exactly the same position in the three snapshots. Details of specific bond lengths, i.e., the Fe‒O and Fe‒S distances in the six CpdI structures, are given in the table at the bottom of Figure 8. The Fe‒O distances are in a narrow range from 1.626 to 1.634Å. These distances match the previously calculated CpdI structures of heme enzymes representing the P450s [2,25,32,33,34,35,36,37,38,39,40,41,42] and peroxidases [72,73,74] well. As seen before, the doublet and quartet spin states are calculated to be within 1 kcal mol^‒1^ for each of the snapshots. Overall, therefore, our calculated CpdI structures with QM/MM of wild-type P450 OleT_JE_ match previously reported structures and electronic configurations well.

The group spin densities of the six CpdI structures are very similar with a spin of 2.06 on the FeO group in the quartet spin state structures, while the doublet spin structures give a value of 2.14. These spin densities, however, are highly polarized toward the iron atom and in the doublet spin the iron spin density is 1.43, while in the quartet spin state it is 1.30. These values contrast gas-phase CpdI calculations that usually find equal spin densities on iron and oxo groups [33]. The rest of the spin density is located on the heme and cysteinate axial ligand. In our particular system, the spin is roughly equally distributed over the heme and cysteinate groups. The results clearly match the previous QM/MM calculations of ours and others on P450 CpdI structures [40,75] that show the valence tautomerization of the mixing of the a_2u_ porphyrin orbital with a lone pair on the axial sulfur ligand.

**Figure 9:**
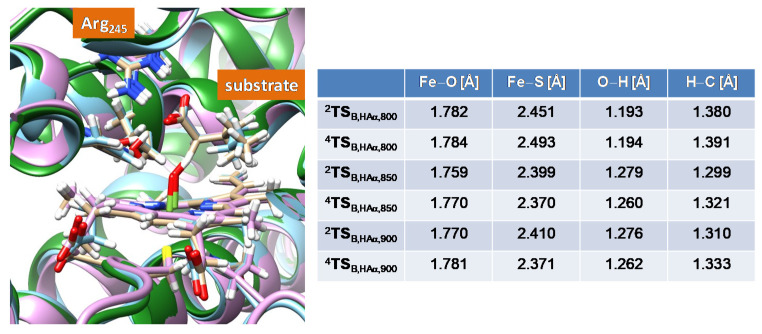
QM/MM optimized geometries of hydrogen atom abstraction transition states from the C^α^‒H position of substrate in the doublet and quartet spin states for snapshots Sn_800_, Sn_850_ and Sn_900_. Fe‒O, Fe‒S, O‒H and H‒C distances given in angstroms, while the left-hand-side gives an overlay of the doublet spin optimized structures.

**Figure 10:**
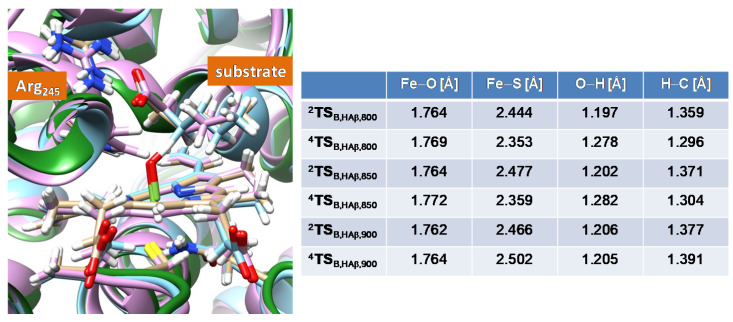
QM/MM optimized geometries of hydrogen atom abstraction transition states from the C^β^‒H position of substrate in the doublet and quartet spin states for snapshots Sn_800_, Sn_850_ and Sn_900_. Fe‒O, Fe‒S, O‒H and H‒C distances given in angstroms, while the left-hand-side gives an overlay of the doublet spin optimized structures.

Thereafter, we investigated the hydrogen atom abstraction steps from the C^α^‒H and C^β^‒H positions of substrate in the doublet and quartet spin states using QM/MM: ^4,2^**TS**_B,HAα_ and ^4,2^**TS**_B,HAβ_. Figure 9 displays extracts of the optimized geometries of the hydrogen atom abstraction transition states from the C^α^‒H position, while the ones for the C^β^‒H position are shown in Figure 10. The overlay of the ^2^**TS**_B,HAα_ structures, similar to the overlay of the reactant complexes, shows that the active site structure of the three transition states is very similar and that little has changed in the position and orientation of the substrate and oxidant. Structurally, the Fe‒O and Fe‒S distances of the six hydrogen atom abstraction barriers from the C^α^‒H positions of substrate range from 1.759–1.784Å and from 2.370–2.493Å, respectively. The hydrogen atom abstraction transition states have the hydrogen atom almost midway between the donor and acceptor groups under and angle O‒H‒C that is close to 180°. In all structures, the O‒H distance is shorter than the C‒H distance, indicating product-type structures. Generally, product-type transition states correspond to lower barriers on the potential energy profile as seen for a series of hydrogen atom abstraction reactions by small model DFT complexes [62,63,76,77]. In particular, the O‒H distances range from 1.193Å for ^4^**TS**_B,HAα,800_ to 1.279Å for ^2^**TS**_B,HAα,850_, while the C‒H distances range from 1.299Å for ^2^**TS**_B,HAα,850_ to 1.391Å for ^4^**TS**_B,HAα,800_. As such, the two spin states and the three snapshots give similar distances and transition state structures for the hydrogen atom abstraction transition states for the C^α^‒H position of hexanoate substrate by P450 OleT_JE_ CpdI.

The group spin densities for the C^α^‒H hydrogen atom abstraction transition states show an increase in spin on the substrate (about -0.45 in the doublet spin and +0.50 in the quartet spin state). As such, a hydrogen atom abstraction takes place and not a proton or a hydride transfer and an electron is transferred from the substrate into the heme.

The hydrogen atom abstraction transition states from the C^β^‒H were calculated for comparison and the optimized geometries in the doublet and quartet spin states for Sn_800_, Sn_850_ and Sn_900_ are given in Figure 10. Similar to the C^α^‒H transition states in Figure 9, also for ^4,2^**TS**_B,Haβ_, the Fe‒O and Fe‒S distances of the optimized geometries fall into a narrow window: Fe‒O distances from 1.762–1.772Å are found and Fe‒S distances from 2.353–2.502Å are calculated. Furthermore, the O‒H distances range from 1.197Å for ^2^**TS**_B,HAβ,800_ to 1.282Å for ^4^**TS**_B,HAβ,850_, whereas the C‒H range gives a value of 1.296Å for ^4^**TS**_B,HAβ,800_ up to 1.391Å for ^4^**TS**_B,HAβ,900_. Structurally, therefore, the hydrogen atom abstraction transition states for the C^β^‒H transition states are very similar and so are the set of C^α^‒H transition states. Indeed, an overlay of the structures shows little changes in the substrate-binding orientation and positioning. The structures also match the previous QM/MM studies of our group well [61].

The group spin densities for the ^4,2^**TS**_B,HAβ_ transition states similar as those for the α-pathway transition states show a built up of radical character on the substrate, while an electron is being transferred from the substrate into the heme manifold.

### 2.4. QM/MM Calculations on the Asn242Arg/Arg245Asn Double Mutant

As discussed above, substrate positioning in P450 OleT_JE_ may be important and drives the reaction to specific products. Indeed, several studies [18,19,20,21,22,23,24,25,] showed that P450 OleT_JE_ reacts with long and linear fatty acids preferentially to form terminal olefins as main products. However, reactions with branched short-chain fatty acids, such as 2-methyl butyrate, gave α-hydroxylation and C^α^‒C^β^ desaturation products, while with 2-methyl-3-phenyl propionate as a substrate α-hydroxylation and decarboxylation products were obtained and no evidence of β-hydroxylation and desaturation was found [25]. As substrate positioning is determined by the position of the active site Arg residue (Arg_245_ in OleT_JE_), we decided to investigate a double mutant structure, where the Arg has moved to an alternative position in the active site. Based on the structure shown above in Figure 1, we decided to move the Arg to position 242 (occupied by an Asn residue in the wildtype enzyme) and hence created the in silico double mutant Asn242Arg/Arg245Asn.

**Figure 11:**
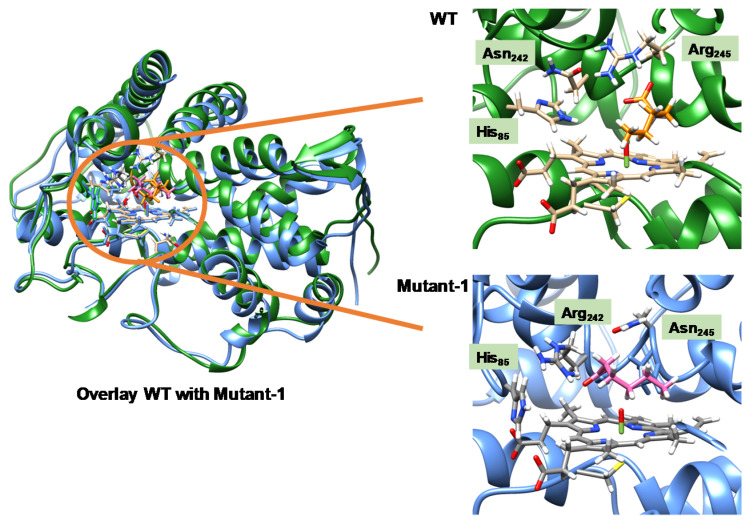
Left: Overlay of the wild-type and Asn242Arg/Arg245Asn double mutant structures (mutant-1) of ^2^CpdI for the snapshot taken after 800ps. Active site structures with key amino acids and substrate (in orange or pink) highlighted. As can be seen, the active site Arg residue guides and positions the substrate in the binding pocket.

Figure 11 shows an overlay of the wild-type and double mutant (Asn242Arg/Arg245Asn, mutant-1) structures of the doublet spin CpdI structure obtained from snapshot Sn_800_. The other snapshots in the doublet and quartet spin state show similar binding poses and orientations. As can be seen, the double mutation has little effect on the overall fold and secondary structure of the enzyme. Most loops and chains are virtually in the same position. There are, however, dramatic differences in the substrate-binding pocket, which is highlighted in the individual structures on the right-hand side in Figure 11. Thus, in both cases, the substrate carboxylate group forms a salt bridge with the active site Arg residue. In the wild-type structure the C^α^, C^β^ and C ^γ^ positions of the substrate are brought closest to the heme, and, consequently, substrate activation is likely to happen on one of these carbon atoms.

In the double mutant structure, by contrast, the Arg side chain is located at a lower position in the active site and the substrate bends differently, with the C^α^‒H positions point upwards, while the C^β^‒H positions point down, i.e., in the direction of the heme. As such, we predicted more likely C^β^‒H activation of the substrate and decided to test this with a series of QM/MM calculations on the double mutant (Asn242Arg/Arg245Asn, mutant-1) in three different snapshots (Sn800, Sn850 and Sn900). All structures connected to the Asn242Arg/Arg245Asn double mutant are identified with the label M1 in subscript after the name of the structure.

**Figure 12:**
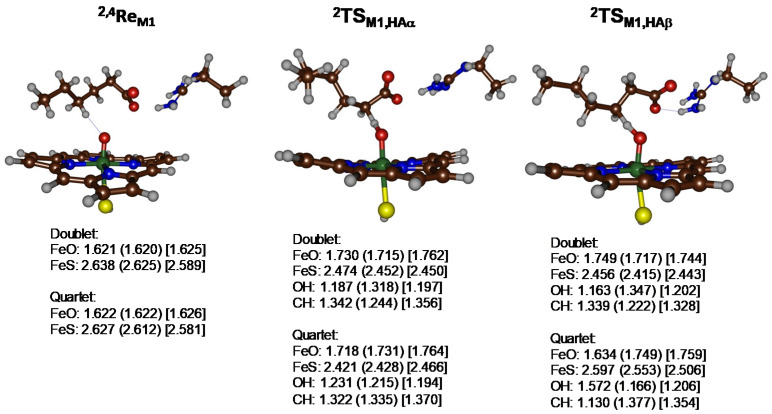
Extracts of the QM region of the QM/MM optimized geometries of the reactant and transition states for C^α^‒H and C^β^‒H hydrogen atom abstraction for the double mutant Asn242Arg/Arg245Asn. Bond lengths are in angstroms for Sn_800_ (Sn_850_) and [Sn_900_].

Optimized geometries of ^2,4^CpdI (^2,4^**Re**_M1_) and the C^α^‒H and C^β^‒H hydrogen atom abstraction transition states (^2,4^**TS**_M1,HA_) for the Asn242Arg/Arg245Asn double mutant are given in Figure 12. The reactant structures have short Fe‒O distances typical of a double bond with values ranging from 1.620 to 1.626Å. These values are slightly shorter than those seen for the wild-type complexes, although this may not be significant. The iron-sulfur distances range from 2.581 to 2.638Å for the six reactant complexes of the double mutant and are somewhat longer than the values for the wild-type system (see Figure 8 and Figure 12). They are also somewhat longer than those normally found for DFT cluster models, where typically values of around 2.4Å are calculated [33]. This probably has to do with the local environment and hydrogen bonding interactions in the active site that weaken the Fe‒S interaction.

Next, we investigated the hydrogen atom abstraction pathways from the C^α^‒H and C^β^‒H positions of the substrate by the double mutant and optimized geometries are shown in Figure 12. Similar to what is seen for the wild-type structures, the Fe‒O interaction in the transition states elongates to 1.715–1.764Å in the **TS**_M1,HAα_ structures, while the Fe‒S distance shortens to 2.421–2.466Å for the six transition states. In Sn_800_ and Sn_900_ the transition state is slightly product-type with almost slightly shorter O‒H than C‒H distances for the transferring hydrogen atom. By contrast, the C^α^‒H transition state for Sn_850_ in the doublet spin state has a longer O‒H than C‒H bond (1.318 vs 1.244Å), whereas in the quartet spin states it is the other way around (1.215Å for the O‒H and 1.335Å for the C‒H interaction). For the **TS**_M1,HAβ_ structures in the doublet spin state, the structures are product-like in Sn_800_ and Sn_900_, while they are reactant-like in Sn_850_. Overall, the structures look typical for hydrogen atom abstraction barriers and compare well with those of the wild-type and earlier gas-phase DFT models [61,62,63,].

Energetically, a Boltzmann weighted average over the three snapshots for the transition state energies (UB3LYP/BS1:Charmm//UB3LYP/BS2 level of theory) of the ^2^**TS**_M1,HAα_, ^4^**TS**_M1,HAα_, ^2^**TS**_M1,HAβ_ and ^4^**TS**_M1,HAβ_ are 15.6, 17.5, 17.2 and 12.1 kcal mol^‒1^ above reactants, respectively, as calculated for the mutant reaction pathways. These energetics show that a small preference of C^β^‒H hydrogen atom abstraction over C^α^‒H hydrogen atom abstraction is predicted for the Asn242Arg/Arg245Asn double mutant of P450 OleT_JE_ using QM/MM. For the wild-type system [61], we previously calculated barriers of 6.7, 9.4, 8.7 and 10.7 kcal mol^‒1^, respectively, for ^2^**TS**_B,HAα_, ^4^**TS**_B,HAα_, ^2^**TS**_B,HAβ_ and ^4^**TS**_B,HAβ_. These values are somewhat lower than the mutant energies reported here because zero-point energy (ZPE) corrections were included in the wild-type calculations, which are known to lower hydrogen atom abstraction barriers by around 3 kcal mol^‒1^ [78,79]. We attempted calculating ZPE values on the QM region of the QM/MM optimized structures by doing a single-point calculation and frequency in Gaussian-09 on the QM region only; however, this gave multiple imaginary frequencies and hence made the data unreliable. Nevertheless, the mutations seem to raise the barriers slightly with respect to the wild type, probably because of the different orientations of the substrate versus oxidant. Thus, the most favorable pathway as obtained with QM/MM for fatty acid activation by wild-type P450 OleT_JE_ is, therefore, C^α^‒H hydrogen atom abstraction, which would lead to α-hydroxylation products. Nevertheless, since the hydrogen atom abstraction barriers from the C^β^‒H position in the wild type are close to those of the C^α^‒H, a mixture of products originating from hydrogen atom abstraction from the C^α^‒H and C^β^‒H positions is predicted as indeed observed experimentally [18,19,20,21,22,23,24,25].

In the mutant structures, by contrast to the wild type, the lowest barrier is for the ^4^**TS**_M1,Haβ_ pathway and, as such, we would expect C^β^‒H hydrogen atom abstraction as the major reaction channel for the Asn242Arg/Arg245Asn double mutant of P450 OleT_JE_. If C^β^‒H hydrogen atom abstraction is followed by a quick decarboxylation step, as seen in our previous QM/MM studies [55,61], this would imply a regioselectivity change to a dominant decarboxylation pathway. Consequently, our QM/MM studies predict that the Asn242Arg/Arg245Asn double mutant of P450 OleT_JE_ will change the positioning of the substrate in the substrate-binding pocket and may lead to a change in substrate activation and generate a lesser amount of α-hydroxylation and possibly more decarboxylation products. It would be interesting to see if this double mutant indeed gives the predicted product distributions.

## 3. Materials and Methods

### 3.1. Computation

The calculations were split into two sets, whereby, initially, small model complexes of the active site of the enzyme were investigated in order to obtain the “ideal” geometry of the transition states for substrate decarboxylation. These structures were then inserted into the substrate-bound protein databank (pdb) file and based on the substrate orientation attempts were made to bioengineer the substrate-binding pocket by moving the active site Arg residue that forms a salt bridge to the fatty acid carboxylate to another position. Thereafter, a series of quantum mechanics/molecular mechanics (QM/MM) studies were performed on the mechanisms of substrate decarboxylation versus hydroxylation by a CpdI model.

### 3.2. Model Complexes

DFT models were set up of P450 CpdI with fatty acid substrate and the electronic and catalytic properties were investigated. In particular, hydrogen atom abstraction by CpdI from the substrate at the α- and β-positions were studied. A simplified gas-phase model was developed of CpdI as an iron(IV)-oxo porphyrin cation radical with thiolate as the axial ligand, namely [Fe^IV^(O)(Por^+^^∙^)SH] with Por = porphyrin. The substrate was abbreviated to hexanoate and the full mechanisms leading to decarboxylation and α- and β-hydroxylation were investigated.

All DFT modelling was performed on the Gaussian 09 software (Wallingford, CT, USA) [80] with standard convergence criteria at the UB3LYP level of theory [81,82]. The geometry optimizations, frequencies and geometry scans were performed with the 6-31G* basis set, except for iron, where a LANL2DZ basis set with electron core potential (ECP) was employed [83,84]. Frequency calculations were done for all optimized structures and gave only real vibrations for local minima and a single imaginary mode for the transition states across the transferring reaction coordinate. Single-point energies calculated for all structures with an implicit solvation model (continuum polarized conductor model) [85] with a dielectric constant mimicking water) at the 6-311+G* level of theory on all atoms, plus an LACV3P+ basis set with core potential on iron. The quartet spin structures had S^2^ values of around 3.79, which is close to the theoretical value of 3.75, hence we considered the unrestricted DFT formalism suitable for these systems.

### 3.3. Enzyme Set-Up

In the next stage of the project, QM/MM structures were set up based on the 4L40 protein data bank structure [31], using procedures reported previously [65,86,87,88]. The wild-type structure was converted from an iron(III)-heme with ligated water molecule into an iron(IV)-oxo(heme+•) species by removing the water protons and changing the Fe‒O distance to 1.63Å. Subsequently, the protein was protonated at pH 7, using the PDB2PQR web service [70], whereby all carboxylates (Glu/Asp) were deprotonated and all Arg and Lys residues protonated. The His residues were visually inspected for proton–donor and proton–acceptor interactions and all histidine residues were taken as singly protonated. The substrate was placed in position on the wild-type crystal by the SwissDock web service (Lausanne, Switzerland) [89].

In the next stage, bioengineered models were created by making in silico mutations, where the salt bridge between substrate and active site Arg was moved to another position. In particular, the double mutant Asn242Arg/Arg245Asn was investigated in detail. Although the wild type was studied previously [61], we redid the model set up for consistency using the same level of theory and convergence criteria as for the mutant systems.

The subsequent set-up, equilibration and solvation of WT and double mutant structures were done with the CHARMM software package using the Charmm force field [71]. The substrate topology and parameter files for CHARMM were produced by the Swissparam web service. All starting structures were energy minimized, solvated by a sequence of 20 solvation-equilibration cycles, and neutralized by counter-ions and heated to room temperature prior to subject to a 1 ns molecular dynamics (MD) simulation. The MD simulations converged with stable root-mean-square-deviation (RMSD) values after a few 100 ps and several snapshots were selected as starting structures for the QM/MM calculations. A total of three snapshots were taken from each system, namely after 800, 850 and 900 ps, designated Sn_800_, Sn_850_, and Sn_900_.

### 3.4. QM/MM Calculations

Subsequently, a previously reported methodology for QM/MM modelling was employed [61,64,65,66,67,90], run through a combination of Turbomole [91] and the DL-Poly [92] (using CHARMM Force Field (Harvard, Cambridge, MA, USA) [71]) interface as provided by the Chemshell software [93]. Thus, Turbomole describes the QM region with DFT methods and DL-Poly the MM region using Charmm. In all calculations, the QM/MM calculations were performed with the unrestricted B3LYP density functional method in combination with an SVP basis set on all atoms [94]. To improve the energies, a single-point calculation with a larger basis set, namely def2-TZVP on all atoms, was performed [95]. All calculations were done for the doublet and quartet spin states. These methods have previously been applied for the studies of enzymatic reaction mechanisms and shown to reproduce experimental rate constants, regioselectivities and kinetic isotope effects well [96,97,98,99,100,101].

The smallest QM region (**A**) was described by the heme (without substituents) and included the iron(IV)-oxo group, thiolate for Cys_365_, the side chain of Arg_245_ and the hexanoate substrate. Bonds on the border between the QM and MM region are highlighted with wiggly lines and were described with the Link-atom method. Moreover, for the WT structure, a more elaborate QM region (**AB**) was investigated that, in addition to QM region **A**, consisted of the Asn_242_ and His_85_ side chains and three water molecules.

Previously, we validated our computational methods by testing several density functional theory methods for P450 OleT_JE_ calculations [61]. Thus, after the initial QM/MM calculations at the UB3LYP/BS1 Charmm level of theory, a series of single-point calculations at the UPBE0/BS2 [102] and UB3LYP-D3/BS2 [103] level of theory were performed. These studies [61] predicted the hydrogen atom abstraction transition states from the C^α^ ‒H and C^β^‒H positions of substrate to be within a couple of kcal mol^‒1^ and gave the same spin-state orderings and electronic configurations; hence, they predicted the same trends. Moreover, geometry optimizations with alternative density functional methods gave similar structures and analogous spin-state orderings; therefore, we are confident that the methods are reliable. For analogous iron-porphyrin complexes and particular Cpd I models, we tested various computational methods and techniques. Thus, geometries optimized at UB3LYP/BS1, UB3LYP/BS2 and UB3LYP-D3/BS2 give virtually the same spin-state ordering and very similar geometries [53] and hence this is not expected to change for the models described here. Further test calculations with a different amount of exchange or correlation included also showed little differences to the obtained results [73].

## 4. Conclusions

In this work, we report a computational study into the P450 peroxygenase OleT_JE_, which binds long-chain fatty acids and generally reacts to form terminal olefins, hydroxylated fatty acids and desaturated fatty acids. We initially used small model complexes of the heme and substrate only and investigated pathways leading to products, which showed that most secondary C‒H bonds can be activated by the active species of the enzyme, i.e., CpdI, with similar reaction barriers. Subsequently, we explored a full enzyme model with QM/MM and showed that substrate positioning affects the substrate activation dramatically. We then created a double mutant structure in silico, i.e., the Asn242Arg/Arg245Asn double mutant, where the positions of the active site Asn and Arg residues are swapped. This mutant positions the substrate differently and the QM/MM calculations predict enhanced C^β^‒H substrate activation, which could lead to higher amounts of terminal olefin products. Our predictions give suggestions for a novel biofuel enzyme system with higher activity and selectivity than wild-type P450 OleT_JE_.
